# CDK12/13 Inhibition Induces DNA Hypomethylation and Sensitizes Cancer Cells to Compounds Targeting DNA‐PK

**DOI:** 10.1111/jcmm.71323

**Published:** 2026-08-20

**Authors:** Jing Liang, Aishwarya Gondane, Harri M. Itkonen

**Affiliations:** ^1^ Department of Biochemistry and Developmental Biology, Faculty of Medicine University of Helsinki Helsinki Finland; ^2^ Department of Clinical Molecular Biology, EpiGen Institute of Clinical Medicine, University of Oslo Oslo Norway; ^3^ EpiGen, Medical Division Akershus University Hospital Lørenskog Norway

**Keywords:** cyclin‐dependent kinase 12, DNA‐dependent protein kinase, epigenetic plasticity, transcription elongation

## Abstract

Inactivation of the major transcription elongation kinase, cyclin‐dependent kinase 12 (CDK12), characterizes a subset of aggressive prostate cancers but it is not known how cells adapt and even benefit from this event. We show that mutations in the *CDK12* gene are associated with decreased DNA methylation in patient tumours and dual inhibition of the major transcription elongation kinases, CDK12 and CDK13 decreases DNA methylation in vitro. This decrease in DNA methylation occurs particularly in the target genes of MYC (MYC Proto‐Oncogene). Previously, it was established that CDK12 inhibition impairs homologous recombination, while increase in MYC activity causes DNA replication stress. We therefore hypothesized that CDK12/13 inhibition renders cells dependent on high DNA‐PK activity, which is required for non‐homologous end joining. Indeed, we show that genetic and pharmacological targeting of DNA‐PK is toxic to cancer cells in combination with CDK12/13 inhibition. In brief, here we show that decrease in CDK12/13 activity depletes DNA methylation from MYC target genes and propose that *CDK12* inactivation may serve as a biomarker of sensitivity to DNA‐PK inhibitors.

**Abbreviations**
AR: Androgen receptor
CDK12: Cyclin‐dependent kinase 12
CRPC: Castration‐resistant prostate cancer
DEGs: differentially expressed genes
DNA‐PK: DNA‐dependent protein kinase
DPF1: Double PHD Fingers 1
DS: DNA double strand
DTT: dithiothreitol
FBS: foetal bovine serum
IDH1: Isocitrate dehydrogenase 1
MeDIP‐Seq: Methylated DNA immunoprecipitation sequencing
PARP: Poly [ADP‐ribose] polymerase 1
PBS: Phosphate‐buffered saline
PC: Prostate cancer
PLCA8: Placenta specific 8
pTEFB: The positive transcription elongation factor
PTEN: Phosphatase and tensin homologue
RNA Pol II: RNA polymerase II
RTNL1: Reticulon 4 receptor
SLAM‐DUNK: Thiol(SH)‐linked alkylation for the metabolic sequencing of RNA‐Digital Unmasking of Nucleotide conversions in K‐mers
SLAM‐seq: Thiol(SH)‐linked alkylation for the metabolic sequencing of RNA
TDRD1: Tudor Domain Containing 1

## 1 Background

Prostate cancer (PC) is among the most commonly diagnosed cancers in men in the US, and currently, the therapy‐induced castration‐resistant PC (CRPC) has no curative measures [1, 2]. CRPC cells rewire their transcriptomes to allow continuous proliferation despite the presence of anti‐androgens [3]. Cancer cells typically exhibit high levels of transcription, and it is therefore unexpected that inactivation of cyclin‐dependent kinase 12 (*CDK12*), a transcription elongation kinase, is associated with a more aggressive cancer phenotype [3, 4].

CDK12 phosphorylates RNA polymerase II (RNA Pol II) to promote transcription elongation of the particularly long genes [5, 6]. Many of these genes are involved in DNA repair, particularly in homologous recombination, and targeting CDK12 can sensitize cancer cells to PARP inhibition [7, 8]. RNA Pol II carboxy‐terminal domain is target for the dynamic phosphorylation events, and CDK12 maintains the phosphorylation status along the long genes to support productive elongation [5, 6, 9, 10, 11]. CDK12 and CDK13 are structurally related kinases and exhibit partial redundancy in their function, and to efficiently block transcription elongation, one must target both [10, 11]. Dysregulation of CDK12 activity has been implicated in various cancers, including prostate cancer [12], breast cancer [13] and ovarian cancer [14].

*CDK12* gene is mutated and/or deleted in a relatively high frequency in prostate cancer, and this genetic alteration is associated with increased genomic instability. Inactivation of the *CDK12*‐gene is most common in prostate cancer, but it also occurs in ovarian cancer [15]. The most striking effect of CDK12/13 inhibition in vitro is defective expression of the particularly long genes, many of which encode for the DNA repair machinery [5, 6]. Transcriptional changes are apparent when CDK12 activity is acutely depleted but practically nothing is known about the epigenetic landscape reinstated in this situation.

Decrease in CDK12 activity can in several ways positively regulate transcription to confer growth advantage. *CDK12* inactivation is associated with *MYC* amplification [16], potentially due to increase in genomic duplication events when the expression of the DNA repair machinery is impaired. MYC functions as a transcriptional amplifier [17], and enhancement of MYC signalling may be important to counteract the transcriptional defects when CDK12 activity is compromised. MYC is also a major oncogene, aberrant activation of which promotes cell proliferation [18]. In addition to genomic instability, CDK12/13 inhibition also activates the expression of short genes by releasing CDK9 from its inhibitory complex [19]. Evidently, the decline in CDK12 activity leads to drastic effects on transcription; however, it is not known if the transient effects lead to remodelling of the transcription machinery itself.

We set to probe how cells respond to a decline in CDK12 activity using CRPC as our model system because this disease is currently lethal. To assess potential epigenetic effects, we used patient data profiled for DNA methylation, which revealed overall decline in global DNA methylation. Next, we probed the causality between an acute decline in CDK12 activity and decrease in DNA methylation in vitro. Strikingly, the decrease in DNA methylation occurred in MYC binding sites in both patient tumours and cell line experiments. We hypothesized that defective transcription elongation in conjunction with activation of the MYC oncogene renders cells dependent on DNA‐PK, the kinase required for non‐homologous end joining during double‐strand breaks. Indeed, we show that the cells with lowered CDK12 activity become dependent on high DNA‐PK activity to sustain RNA Pol II phosphorylation and MYC‐dependent transcription. Finally, co‐targeting of CDK12/13 and DNA‐PK is combinatorial lethal to prostate, breast and ovarian cancer cells, and we therefore propose that *CDK12*‐inactivation is a biomarker of sensitivity to DNA‐PK inhibitors.

## 2 Materials and Methods

### 2.1 Cell Culture, Proliferation Assays and Western Blotting

C4‐2, 22RV1 and MDA‐MB‐231 cell lines were obtained from the American Tissue Culture Collection, and these cell lines were maintained in RPMI medium supplemented with 10% foetal bovine serum (FBS). COV318 and OVCAR3 cell lines were a kind gift from Professor Liisa Kauppi (University of Helsinki). COV318 was maintained in DMEM high glucose medium supplemented with 10% FBS. OVCAR3 cells were maintained in RPMI medium supplemented with 10% FBS plus 10 μG/mL insulin (Thermo Fisher Scientific 11508856).

Transfection of Ambion Silencer Select siRNAs against PRKDC (Catalogue # 4427038/Assay IDs s773 and s774) and CDK12 (Catalogue # 4392420/Assay IDs s28622 and s28623) was achieved using RNAiMax (Invitrogen Lipofectamine RNAiMAX Transfection Reagent, Catalogue number: 13778150). For viability assays, knockdown was performed for 24 h, after which the indicated treatments were added for 4 days. Next, CellTiter‐Glo 2.0 assay (Promega) was used according to the manufacturer's instructions in technical triplicates in 384‐well plates, and the data presented is from at least three biological replicates.

For Western blot experiments, cells were seeded in 6‐well plates at a density of approximately 300,000 cells per well and allowed to grow for 24 h, after which they were treated as indicated in the figure legends. Cell lysates for western blot were prepared as previously described (except no sonication) [20]. Specifically, following collection in ice‐cold PBS, cells were centrifuged at 3000 rpm for 5 min. All steps were carried out on ice. After washing with PBS, cells were incubated in RIPA buffer (10 mmol/L Tris–HCl (pH 8.0), 1 mmol/L EDTA, 0.5 mmol/L EGTA, 1% Triton X‐100, 0.1% sodium deoxycholate, 0.1% SDS and 140 mmol/L NaCl) with protease and phosphatase inhibitors for a minimum of 30 min to achieve lysis. The lysates were then centrifuged at 14,000 rpm for 5 min and the resulting supernatants were retained for western blot. Protein concentrations were determined using the BCA assay. Antibodies used are from Cell Signaling Technology: PARP (CST9532), p‐S2‐RNA Pol II (13499); from Proteintech: CDK12/CRKRS (26816‐1‐AP); and from Abcam: Actin (ab49900). Compounds were obtained from MedChemExpress. Crystal violet staining assay was used to measure the colony‐formation ability of cells as previously described [21]. Briefly, cells were sequentially fixed in ice‐cold 70% MeOH (2 min) and 100% MeOH (10 min), allowed to air‐dry, and stained with 0.05% crystal violet prepared in 25% MeOH for 10 min. After this, representative images were acquired.

### 2.2 Spheroid Experiments

22RV1 cells were plated in Matrigel (Corning, catalogue number: 354234) and allowed to grow for 1 day before treatment. Spheroids were treated for 7 days with 100 nM THZ531 (CDK12/13 inhibitor), 2 μM AZD7648 (DNA‐PK inhibitor) and combinations. To visualize the spheroids, we imaged them using Zeiss AxioObserver inverted widefield microscope (Plan‐Apochromat 20×/0.8). CellTiter‐Glo 2.0 assay (Promega) was used to lyse the organoids, and the luminescence‐signal was recorded. The data generated is from four biological replicates.

### 2.3 Preparation of Samples for MeDIP‐Seq and qPCR Validation

For MeDIP‐seq, 22RV1 cells were seeded in petri‐dishes and cultured for 1 day. The next day, cells were split into an untreated group and a 150 nM THZ531‐treated group, which were collected after 3 days of treatment. DNA was purified using Wizard Genomic DNA Purification Kit (Promega) according to manufacturer's instructions. After this, the purified DNA was sonicated using the ultrasonic disintegrator (MSE Soniprep 150, settings three amplitude microns for 10 times, 10 s on, 20 s off on ice). The EpiMark Methylated DNA Enrichment Kit (New England Biolabs, catalogue number E2600S) was used to purify double‐stranded methyl‐CpG DNA from the DNA sample according to manufacturer's instructions. For qPCR‐based validation, we used the following primers: AR promoter F‐TCGACCATTTCTGACAACGC and R‐GAAGCTGTTCCCCTGGACTC, MYC promoter F‐GAGATCCGGAGCGAATAGGG and R‐TGGGCAAAGTTTCGTGGATG and TDRD1 promoter F‐ATTGGAGGCGAGGGAAGT and R‐ACCTGGAAAGAGGCAAGAG. qPCR assays were run with PowerUp SYBR Green Master Mix (Thermo Fisher Scientific, catalogue no. A25742) according to the manufacturer's instructions. Subsequently, both the library preparation and sequencing were purchased as service from the BGI (two biological replicates with PE100bp on a DNBseq platform to obtain at least 35 million high quality reads per sample). For differential methylated sites validation, we used the following primers: NR_125885.1 F‐GCTGGGGACTGGGAACTTT and R‐CCAAACAGACACGTCACGC, DPF1 F‐ TTAGGCTTAAGGGGCAGAGG and R‐GTGAACATGACGGCAGCC, RTN4L1 F‐GAAACAGGGGCGGATTAAGG and R‐TCTGCCTCTCCCCTCCCA and PLAC8 F‐GATCATTCAGGGCGTGCATG and R‐CTTCAGACCTGTCCCTGCAA.

### 2.4 SLAM‐Seq

For metabolic labelling experiment (SLAM‐seq), C4‐2 cells were grown in 6‐well plates for one day. The next day, cells were untreated and treated with 20 μM AZD (DNA‐PK inhibitor), 100 nM THZ531 (CDK12/13 inhibitor) and combinational treatment of AZD7648 and THZ531 for 4 h. In the last 10 min prior to collecting cells, they were labelled with 1 mM 4‐thiouridine (4sU, obtained from ThermoFisher Scientific). RNA was purified using the Amersham RNAspin Mini Kit (catalogue number 25050071) according to manufacturer's instructions except 0.1 mM DTT was included in all the buffers. Purified RNA was alkylated as previously described [22]. Finally, library‐preparation and sequencing were purchased as a service from Lexogen. In brief, libraries were prepared using QuantSeq 3′ mRNA‐Seq V2 Library Prep Kit with UDI for Illumina sequencing and sequencing was performed using SR100 High Output sequencing on Illumina NextSeq 2000.

### 2.5 Bioinformatics

For MeDIP‐seq analysis, the raw fastq files were filtered using SOAPnuke [23], and aligned to human genome HG38 using bowtie2 [24]. Samtools was used to convert SAM‐files to BAM‐files [25], and Picard (https://broadinstitute.github.io/picard/) was used to remove PCR duplicates. MACS2 (2.2.6) was used to call peaks [26] and Deeptools [27] was used to generate coverage track files. DiffBind [28] was used to identify the significantly differential methylated loci between different conditions. To annotate and categorize the peaks, we used HOMER [29].

For RNA‐ and SLAM‐seq analysis, the raw fastq files were trimmed using cutadapt [30] and aligned to human genome HG38 using bowtie2 [24], and Samtools was used to convert SAM‐files to BAM‐files [25]. Standard RNA‐seq analysis was performed by summarizeOverlaps [31]. For analysis of the SLAM‐seq data, we used SLAM‐DUNK [32]. Before analyzing the data using SLAM‐DUNK, the length of all of the raw reads were trimmed to 114 bp by trimmomatic [33]. Differential gene expression was performed using DESeq2 [34], and the threshold is *p* < 0.05 and less/more than log_2_ fold change ±1. Fgsea [35] was used to perform gene set enrichment analysis (GSEA). Threshold of padj‐Value < 0.05 was selected to define the significantly enriched hallmark pathways. The heatmap‐, pie‐, violin‐, bar‐, box‐, scatter‐ and volcano‐plots were generated using R4.2.0.

## 3 Results

### 3.1 Mutations in *CDK12* Gene Are Associated With DNA Hypo‐Methylation in Patient Tumours

**FIGURE 1:**
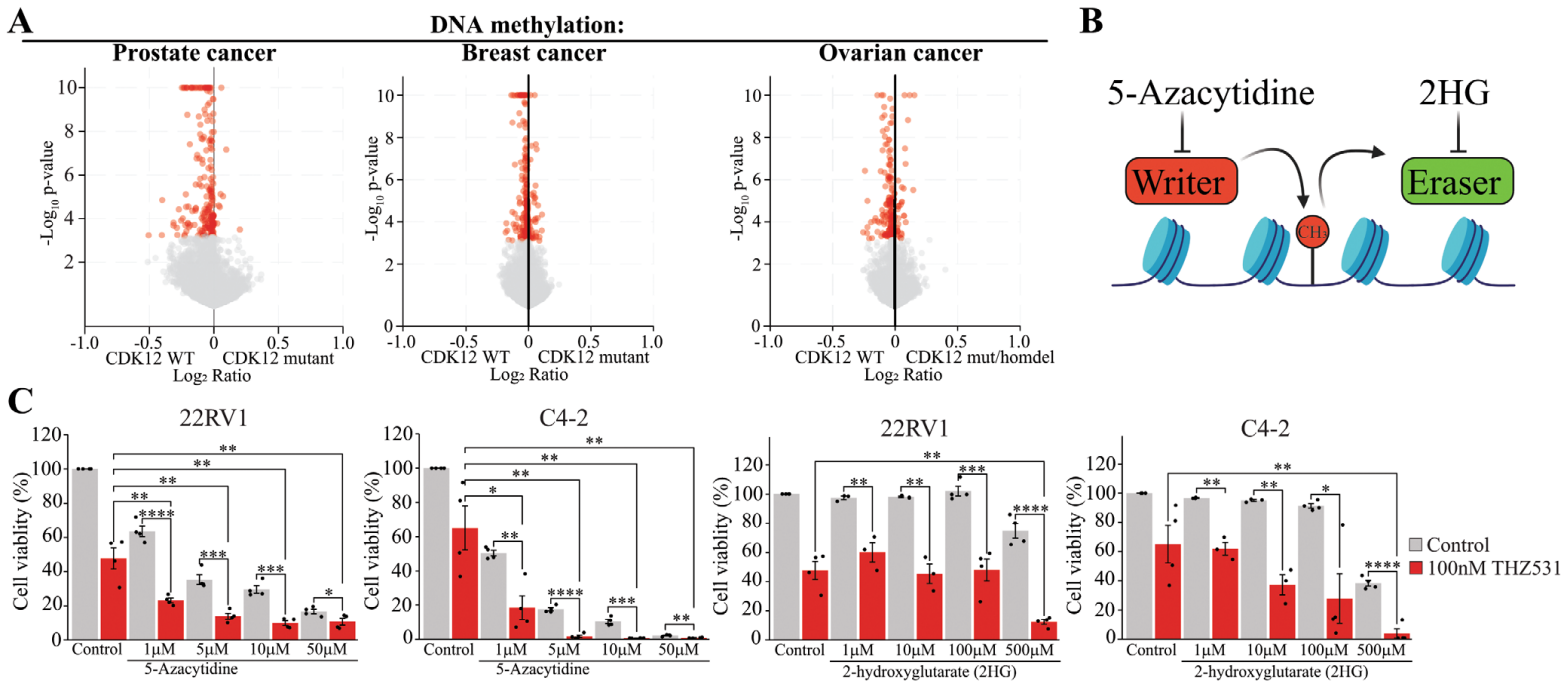
*CDK12* mutations are associated with DNA hypo‐methylation. (A) Prostate and breast cancer tumours with truncating mutations in the *CDK12*‐gene have less DNA methylation than the *CDK12* wild‐type (WT) tumours. Ovarian cancers with either truncating mutations in the *CDK12*‐gene or homozygous deletion of the *CDK12*‐gene have less DNA methylation than the *CDK12* wild‐type (WT) tumours. The plot was generated using data available in the cBioportal, and we used TCGA Firehose Legacy datasets for Prostate Adenocarcinoma, Breast Invasive Carcinoma and Ovarian Serous Cystadenocarcinoma. The x‐axis represents the methylation levels between the two groups. The y‐axis represents the statistical significance, obtained via cBioPortal. Red dots indicate significant differential methylation sites (q‐value < 0.05). Prostate: *CDK12*‐mutated (*n* = 5) and *CDK12* WT (*n* = 487), Breast: *CDK12*‐mutated (*n* = 7) and *CDK12* WT (*n* = 956), Ovarian: *CDK12*‐mutated (*n* = 8) and *CDK12* WT (*n* = 303). Number of significantly affected methylation events is for prostate: WT: 205, *CDK12* mutant: 9, for breast: WT:91, *CDK12* mutant: 41 and for ovarian: WT:153, *CDK12* mutant: 31. (B) DNA methylation can be inhibited using 5‐azacitidine, while DNA demethylation can be inhibited by 2‐hydroxyglutarate. (C) Both 5‐azacitidine and 2‐hydroxyglutarate sensitize castration‐resistant prostate cancer cells (22RV1 and C4‐2 cell lines) to CDK12/13 inhibition. Bar plots present cell viability data (CellTiterGlo) generated from 3 to 4 biological replicates and unpaired Student's *t*‐test was used to confirm the significance (< 0.0001, ****; < 0.001, ***; < 0.01, **; < 0.05, *; no statistical significance reached if nothing is indicated).

We used prostate cancer patient data to probe if inactivation of the *CDK12*‐gene is associated with epigenetic alterations. Curiously, we discovered that truncating mutations in the *CDK12*‐gene are associated with a decrease in DNA methylation in prostate cancer patient samples (The Cancer Genome Atlas, TCGA‐dataset, Figure 1A). The overall magnitude of the effect is modest but clearly many genes show a decrease in DNA methylation. To gain further assurance into the potential association between *CDK12* inactivation and depletion of DNA methylation, we asked if decline in DNA methylation is also found in other cancers. We identified the tumours with truncating mutations and/or deletions in the *CDK12*‐gene in the most common cancer in women (breast cancer) and a cancer previously reported to harbour *CDK12*‐inactivation, ovarian cancer using the TCGA data. Strikingly, *CDK12* truncating mutations in breast cancer patient tumours and *CDK12* mutations and/or deletions in the ovarian cancer patient tumours were associated with a clear decrease in DNA methylation (Figure 1A).

We set out to establish if addition and removal of DNA methylation is important for prostate cancer cells' ability to tolerate a decrease in CDK12 activity (Figure 1B). To target CDK12, we used THZ531, which additionally targets CDK13 [36]. 5‐azacitidine is a clinically‐approved inhibitor of DNA methyltransferases, and the compound causes hypomethylation of DNA [37]. DNA methylation can be induced by treating cells with the oncometabolite 2‐hydroxyglutarate [38]. Both 5‐azacitidine and 2‐hydroxyglutarate significantly sensitized CRPC cells to CDK12/13 inhibition (Figure 1C). Therefore, both addition and removal of DNA methylation are necessary for the CRPC cells' ability to respond to inhibition of CDK12/13. We wanted to probe whether the ability to modify DNA methylation is important to tolerate *CDK12* inactivation in the clinical setting. We hypothesized that this can be probed by identifying the patients that carry specific mutations in the *IDH1‐*gene (isocitrate dehydrogenase 1), which cause a hyper‐methylation phenotype due to accumulation of 2‐hydroxyglutarate, a metabolite inhibiting α‐ketoglutarate‐dependent dioxygenases [39]. Strikingly, truncating mutations in the *CDK12* gene were mutually exclusive with any alterations in the *IDH1*‐gene based on the published available dataset (TCGA, Firehose Legacy, Figure S1). Based on both the patient and cell line data, cells with the lowered CDK12 activity alter their epigenetic landscape through DNA methylation and this process is important for their survival.

### 3.2 Dual Inhibition of CDK12/13 Depletes DNA Methylation in vitro

**FIGURE 2:**
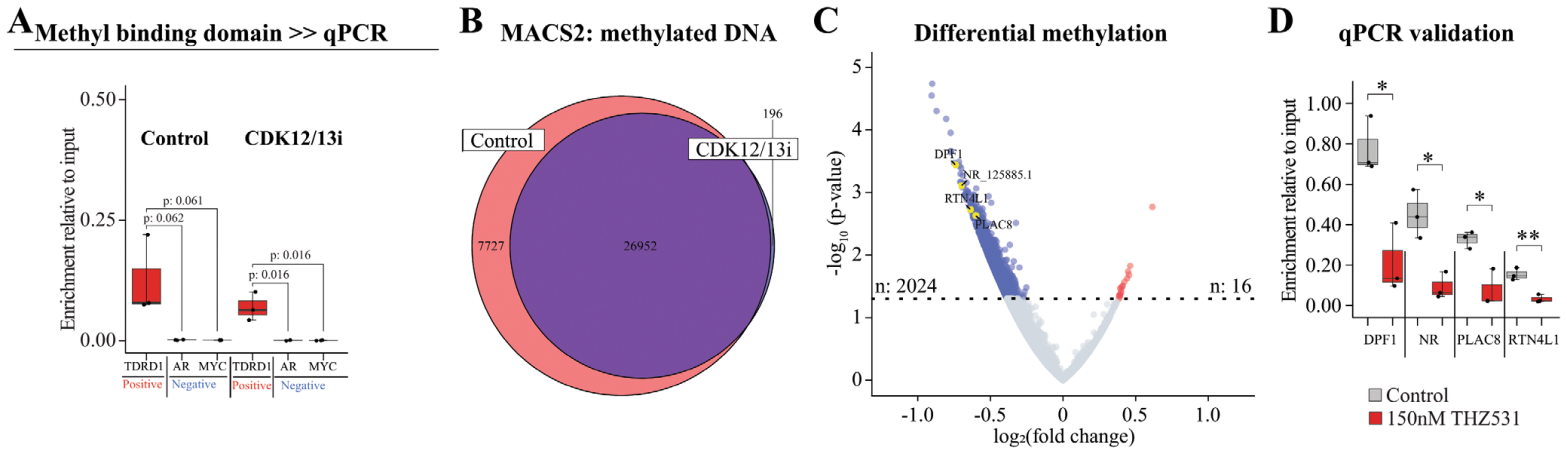
Dual inhibition of CDK12/13 depletes DNA methylation in vitro. (A) Validation of selective enrichment of methylated DNA after MeDIP using qPCR (prostate cancer cell line 22RV1, 150 nM THZ531). Promoter of *TDRD1* is methylated while promoters of *AR* and *MYC* are not methylated as determined using a previous DNA methylation profiling of the same cell line (GSE74464) [40]. Unpaired Student's *t*‐test was used to assess the significance (*n* = 3). (B) Peak calling of methylated sites using MACS2 [26] from untreated (control) and CDK12/13 inhibitor treated cells (150 nM THZ531 for 3 days). (C) We used DiffBind [28] to identify differentially methylated sites between control and CDK12/13 inhibitor treated cells. The genes with significantly (*p* < 0.05) decreased or increased methylation signals are highlighted with blue/yellow and red colours, respectively. (D) Validation of sites that lost DNA methylation using qPCR after 3 days treatment with 150 nM THZ531 (these four genes are coloured by yellow in Figure 2C, three biological replicates and unpaired Student's *t*‐test was used to evaluate the significance: < 0.01, **; < 0.05, *; no statistical significance reached if nothing is indicated). 22RV1 cell line was used in all experiments presented in this figure.

We used methyl binding domain (MBD)‐based enrichment of methylated chromatin to ask how decrease in CDK12/13 activity affects DNA methylation in CRPC cells. For these experiments, we treated cells for three days with CDK12/13 inhibitor to allow potential changes to take place. We note that a significant number of cells die during the treatment, and the remaining population therefore represents cells that can tolerate the lowered CDK12/13 activity. For validation of our MBD‐enrichment, we identified the loci that were reported to be methylated in the same prostate cancer cell line we used here (22RV1) with the same experimental approach [40]. We selected the promoter of *TDRD1*‐gene, which is not expressed in the ERG‐negative prostate cancer cells and 22RV1 cells do not express ERG [41]. For negative control, we used primers against the transcriptionally active promoters of the *AR*‐ and *MYC*‐genes (Figure 2A). We detected at least a 5‐fold significant enrichment between our positive and negative controls (Figure 2A). In agreement with the patient data, inhibition of CDK12/13 activity modestly decreased methylation at the sites assessed, and we went ahead to sequence the MeDIP‐enriched chromatin.

Interestingly, MeDIP‐seq revealed 7727 total number of less methylated sites in response to CDK12/13 inhibition as determined using the MACS2 peak caller (Figure 2B). We wanted to further validate differential methylation in response to CDK12/13 inhibition, and therefore used DiffBind [28] to compute the differential binding sites. This approach identified 2024 significantly lost sites and only 16 gained sites when cells were treated with CDK12/13 inhibitor for three days (Figure 2C). To validate the selective decrease in DNA methylation in response to CDK12/13 inhibition, we selected four genes for qPCR‐based validation based on visual inspection of the data on Integrated Genome Viewer (*NR125885.1*, *DPF1*, *RTN4L1* and *PLAC8*), which all confirmed significant decrease in DNA methylation (Figure 2D). Finally, to confirm that decrease in CDK12 activity alone is sufficient to cause significant impact on DNA methylation, we repeated the MeDIP qPCR‐experiment in 22RV1 and C4‐2 cells after knockdown of CDK12 using two independent siRNAs. This experiment revealed that knockdown of CDK12 significantly decreases DNA methylation on the DPF1‐gene; however, to achieve full impact on DNA methylation, dual inhibition is required (Figure S2).

### 3.3 CDK12/13 Inhibition Depletes DNA Methylation From MYC Binding Sites

**FIGURE 3:**
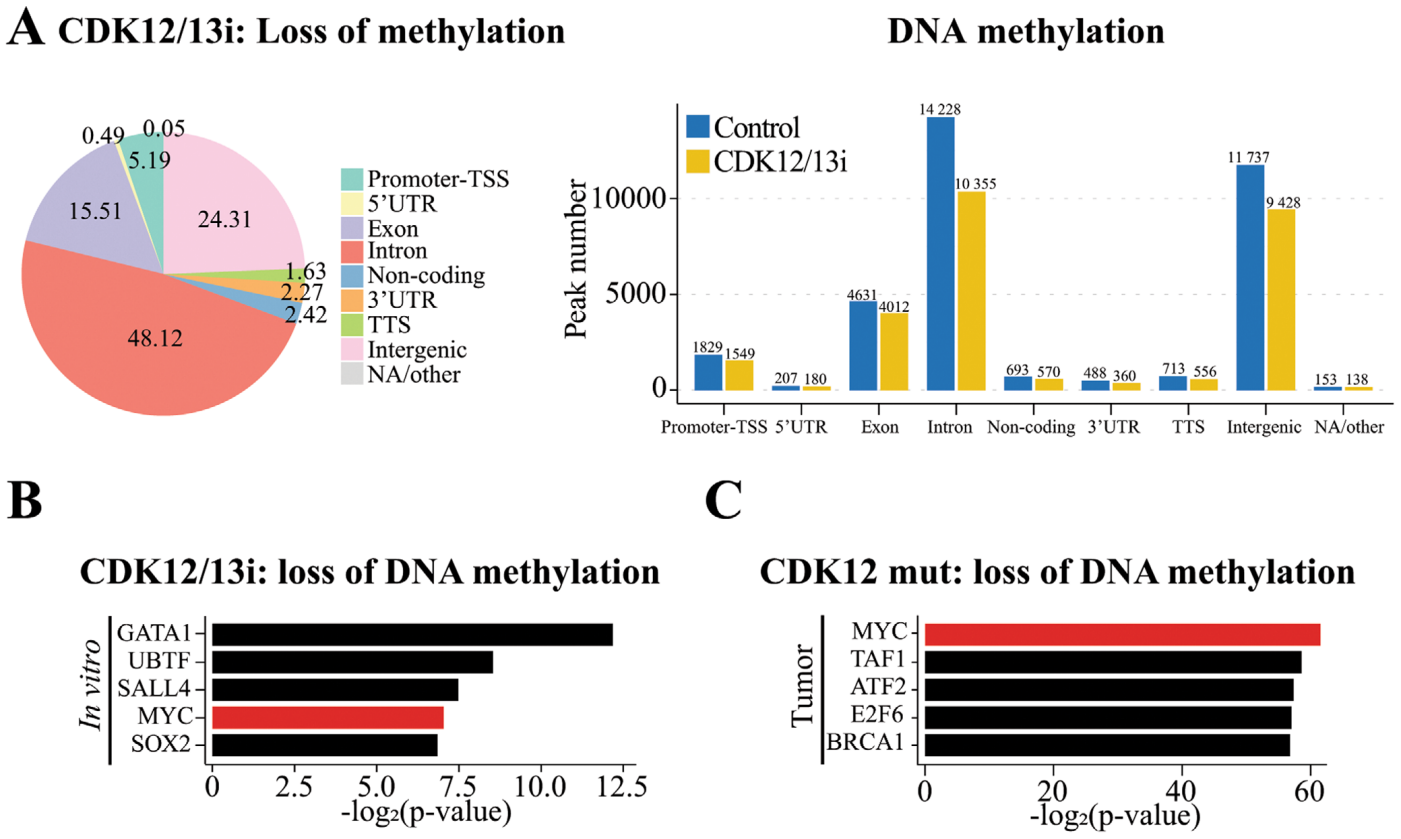
Decrease in CDK12/13 activity depletes DNA methylation from MYC binding sites in vitro (22RV1) and in patient tumours. (A) Left: Annotation of methylation peaks that were identified as hypo‐methylated after CDK12/13 inhibition using THZ531 (CDK12/13i). Right: The enriched peaks of the untreated and the treatment with THZ531 inhibitor. We used Homer [29] to annotate the peaks. The pie chart shows the annotation of sites that lost methylation in percentage (2024 sites from Figure 2C), while the bar‐plot shows the number of total peaks in annotation classification with and without CDK12/13 inhibition. (B) Genes that lose methylation were subjected to further analysis using Enrichr dataset of ENCODE and ChEA Consensus TFs from ChIP‐X. (C) We used ENCODE and ChEA Consensus TFs from ChIP‐X accessible through the Enrichr to identify transcription factors for genes that lost DNA methylation in *CDK12* mutant tumours (TCGA Firehose Legacy dataset of Prostate Adenocarcinoma accessed through the cBioportal).

To better understand where the loss of DNA methylation occurs when CDK12/13 is inhibited, we annotated the total peaks and differentially methylated peaks based on chromatin features. This revealed that CDK12/13 inhibition decreases DNA methylation predominantly on introns and only a small fraction occurs on the transcription start sites/promoters (Figure 3A). The location along the gene body is consistent with the roles of CDK12/13 as transcription elongation kinases. In addition, we also note robust effects in the intergenic regions; however, this category represents most of the human genome, and it is therefore difficult to study the functional importance of the effect.

We used the ENCODE and ChEA consensus TFs from ChIP‐X to identify the transcription factors that are known to regulate the expression of the genes that lose DNA methylation after CDK12/13 inhibition. This approach revealed an enrichment for GATA1, UBTF, SALL4, MYC and SOX2 (Figure 3B). Next, we applied the same strategy for the sites that lose DNA methylation in prostate cancers that harbour truncating mutations in *CDK12*. Strikingly, the MYC transcription factor was the top‐most enriched factor (Figure 3C). These data identify MYC as a privileged transcription factor gaining access to chromatin when CDK12 activity falls in prostate cancer cells. Earlier, we have shown that inactivation of *CDK12* augments MYC activity in prostate cancer cells [42], and the data presented here propose that this is, at least in part, due to remodelling of the DNA methylation landscape.

### 3.4 Decrease in CDK12 Activity Sensitizes Prostate Cancer Cells to DNA‐PK Inhibition

**FIGURE 4:**
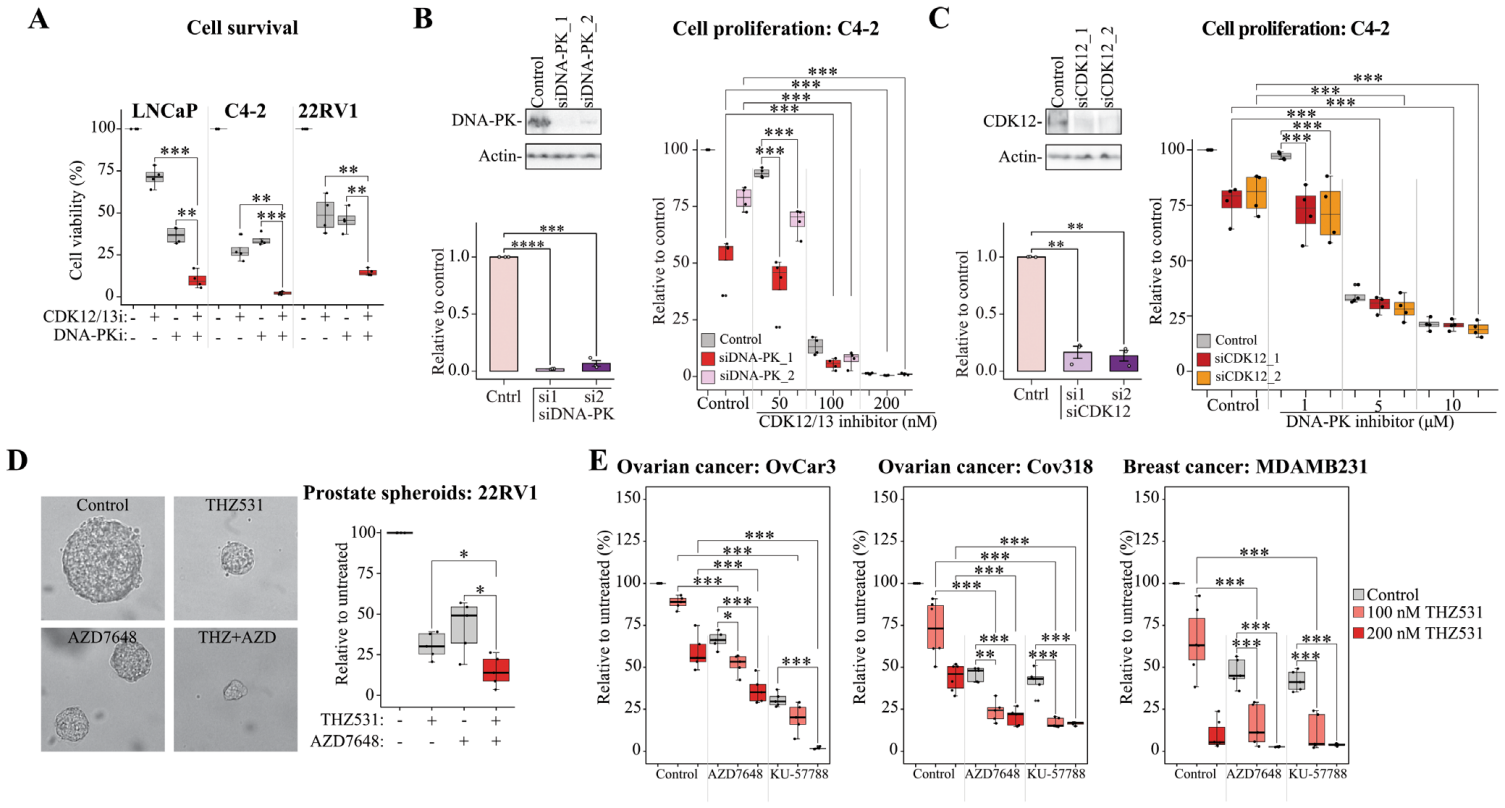
CDK12/13 Inhibition sensitizes cancer cells to DNA‐PK inhibition. (A) Combined targeting of CDK12/13 (100 nM THZ531) and DNA‐PK (5 μM AZD7648) is toxic to androgen‐dependent (LNCaP) and androgen‐independent (C4‐2 and 22RV1) prostate cancer cell lines (4 biological replicates, each 3 technical replicates and independent samples *t*‐test was used to assess the significance). Faint vertical bars visually separate different cell lines. (B) Knockdown of DNA‐PK sensitizes C4‐2 cells to CDK12/13 inhibitor THZ531. Confirmation of DNA‐PK knockdown using western blotting. Densitometry analysis of DNA‐PK protein levels relative to Actin confirms its successful knockdown (cells were treated for 3 days; *n* = 3 biological replicates; statistical significance was determined using *t*‐test). Cell viability after 4 days of DNA‐PK knockdown with CDK12/13 inhibition (THZ531) for the last three days. The data is from four biological replicates (each having three technical replicates) with standard error of mean, and ANOVA was used to assess the significance. Faint vertical bars visually separate different treatment doses of CDK12/13 inhibitor. (C) Knockdown of CDK12 sensitizes C4‐2 cells to DNA‐PK inhibitor AZD7648. Confirmation of CDK12 knockdown using western blot (the rest as in 4B). Densitometry analysis of CDK12 protein levels relative to Actin confirms its successful knockdown (cells treated for 3 days; *n* = 3 biological replicates; statistical significance was determined using *t*‐test). The methods for collecting and statistically testing the cell viability results are the same as those for 4B. Faint vertical bars visually separate different treatment doses of DNA‐PK inhibitor. (D) Spheroid culture (22RV1) confirms that depletion of CDK12/13 activity sensitizes prostate cancer cells to DNA‐PK inhibition (100 nM THZ531 and 2 μM AZD7648). Representative images of cells grown in Matrigel for 7 days. Spheroids were lysed in CellTiterGlo‐reagent, signal recorded and normalized to the signal of the untreated spheroids (4 biological replicates with SEM and independent samples *t*‐test was used to assess the significance). (E) Depletion of CDK12/13 activity using THZ531 sensitizes ovarian and breast cancer cells to DNA‐PK inhibition (5 μM AZD7648 or 5 μM KU‐57788). Cells were treated as indicated for 4 days and cell viability was assessed using CellTiterGlo (4 to 6 biological replicates, each three technical replicates). ANOVA was used to assess the significance: < 0.001, ***; < 0.01, **; < 0.05, *; No statistical significance reached if nothing is indicated. Faint vertical bars visually separate the different DNA‐PK inhibitor treatments.

We hypothesized that inhibition of CDK12/13 leads to dependency on non‐homologous end‐joining to survive stress imposed by defective transcription of genes involved in homologous recombination in the presence of the increased MYC activity. To directly assess if prostate cancer cells become dependent on non‐homologous end‐joining when CDK12/13 is inhibited, we obtained DNA‐PK inhibitor AZD7648. AZD7648 was described in 2019 [43], and the compound has translational value as it has progressed to a clinical trial (NCT03907969). CDK12/13 inhibitor THZ531 sensitized both prostate cancer (LNCaP) and CRPC (C4‐2 and 22RV1) cell lines to AZD7648 (DNA‐PK inhibitor) as determined using cell viability and colony formation assays (Figures 4A and S3). Any small molecule inhibitor can have off‐target effects, and we therefore also used siRNAs to knockdown DNA‐PK and CDK12. Indeed, knockdown of DNA‐PK sensitized prostate cancer cell lines to CDK12/13 inhibitor THZ531 (Figures 4B and S4). Reciprocally, knockdown of CDK12 sensitized prostate cancer cell lines to DNA‐PK inhibitor AZD7648 (Figures 4C and Figure S5). Spheroid tissue culture better models the structural and functional complexity of the in vivo tumour [44], and this system retains both the epigenomic and the transcriptomic features with the patient tumours [45]. These characteristics establish spheroids as an excellent tool for validation of candidate therapies. Targeting CDK12/13 with THZ531 significantly sensitized prostate cancer spheroids to the DNA‐PK inhibitor AZD7648 (Figure 4D).

Earlier, we revealed that remodelling of DNA methylation is a common feature between prostate, breast and ovarian cancers that have defects in the *CDK12*‐gene (Figure 1A). We therefore hypothesized that also breast and ovarian cancer cells should become dependent on DNA‐PK activity when CDK12/13 is inhibited. Indeed, co‐targeting of CDK12/13 and DNA‐PK with small molecule inhibitors significantly more than the single treatments decreased cell viability of breast cancer cell line and two different ovarian cancer cell lines (Figure 4E). To establish if the combinatorial efficacy is additive or synergistic, we performed synergy calculations. Indeed, the combinatorial targeting of DNA‐PK and CDK12/13 shows synergistic interaction in both prostate cancer and breast cancer cell lines (Figure S6).

### 3.5 Targeting DNA‐PK Prevents CDK12/13 Inhibition‐Induced MYC‐Signalling

**FIGURE 5:**
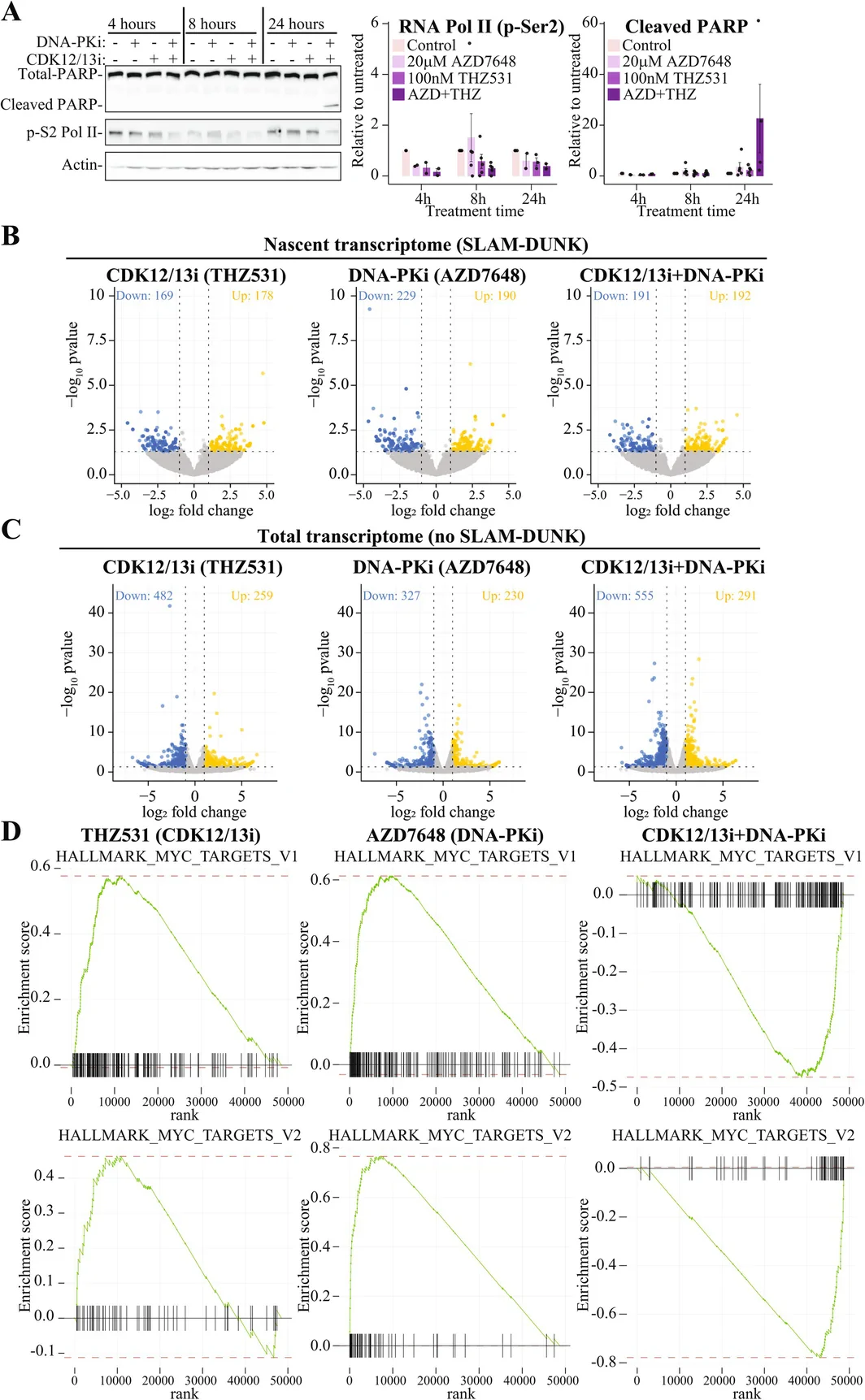
High DNA‐PK activity is required to sustain CDK12/13 inhibition‐induced MYC‐driven transcription. (A) C4‐2 cells were treated with DNA‐PK inhibitor (20 μM AZD7648) and CDK12/13 inhibitor (100 nM THZ531) for 4 h, 8 h and 24 h and samples were analysed using western blot. Densitometry was used to record signal intensity, which is presented relative to control (control is set to 1, and the signal from different treatment samples was first normalized to Actin, n: 2–5). (B) Analysis of the nascent transcription (SLAM‐DUNK [32]) after 4 h treatment with 20 μM AZD7648 (DNA‐PK inhibitor), 100 nM THZ531 (CDK12/13 inhibitor) and combination of AZD7648 with THZ531. (C) Analysis of the same SLAM‐seq data as in 5B using traditional RNA‐seq pipeline and omitting the SLAM‐DUNK step. The genes decreased or increased significantly (*p* < 0.05) and less/more than log_2_ fold change ±1 are highlighted with blue and yellow colours, respectively. (D) Gene set enrichment analysis of the RNA‐seq data (same as in 5C) identifies MYC targets as a gene set that is significantly downregulated in response to combinational inhibition of CDK12/13 and DNA‐PK, while either single inhibition of DNA‐PK or CDK12/13 resulted in significant activation of MYC targets (see also Figure S8). Statistical test used: Kolmogorov–Smirnov test.

DNA‐PK is known to positively regulate pro‐proliferative transcriptional networks in prostate cancer cells through androgen receptor [46, 47]; however, it's role in the context of CDK12/13 inhibition is not known. As the first measure to probe if DNA‐PK is important to maintain RNA Pol II activity when CDK12/13 activity decreases, we evaluated the effects of the combined treatment with CDK12/13 and DNA‐PK inhibitors on RNA Pol II phosphorylation. Indeed, combinatorial treatment decreased the levels of transcriptionally active, Ser2‐phosphorylated RNA Pol II already at 4 h after the treatment in two different CRPC models (Figures 5A and S7). Importantly, the effect on RNA Pol II took place prior to activation of cell death, which became evident only after 24 h of the treatment as determined using PARP cleavage (Figures 5A and S7).

To directly test the hypothesis that combined inhibition of CDK12/13 and DNA‐PK suppresses active transcription, we performed nascent transcriptome profiling using SLAM‐seq after a short treatment time of four hours. In SLAM‐seq, a nucleotide analogue 4‐thiouridine is added to cells for a short duration of time, which enables subsequent computational detection of the newly transcribed mRNAs [22, 32]. In contrast to our hypothesis, combined inhibition of CDK12/13 and DNA‐PK did not further suppress nascent transcription on the global scale (Figure 5B). At a loss of how the combined inhibition of CDK12/13 and DNA‐PK affects transcription, we evaluated the effect on the overall transcriptional programme by analyzing the SLAM‐seq data using the traditional RNA‐seq pipeline as we have performed previously for other treatment combinations [42, 48]. In this case, we noted the highest number of decreased mRNAs for the combination treatment (Figure 5C). This means that combined inhibition of CDK12/13 and DNA‐PK affects the relative abundance of specific mRNAs. We hypothesized that cells switch on/off a specific transcriptional programme when both CDK12/13 and DNA‐PK are inhibited.

To identify this potential transcriptional programme, we used gene set‐enrichment analysis. Curiously, the MYC target genes V2 and V1 were the top‐most significantly downregulated gene sets for the combination treatment, while they were significantly stimulated by the single compound treatments (Figures 5D and S8). In brief, we show that the decrease in CDK12 activity leads to DNA hypo‐methylation, which allows increased activity of the MYC transcription factor and forms targetable vulnerability against DNA‐PK inhibitors.

## 4 Discussion

Here, we report that inactivation of the major transcription elongation kinase, *CDK12*, depletes DNA methylation and went on to model this response in vitro to develop a candidate therapy. We discovered that mutations in the *CDK12*‐gene are associated with decrease in DNA methylation in patient samples (Figure 1A). These data reveal a modest but significant decrease in DNA methylation. We hypothesized that this effect is due to decrease in transcription elongation, and by decreasing DNA methylation, cells can adapt to the defect in the major transcription elongation kinase CDK12.

Other mutations present in the *CDK12* mutant tumours may also contribute to the methylation changes; however, this effect is likely modest because loss‐of‐function mutations or homozygous deletion of *CDK12* are not enriched with other prostate cancer‐relevant mutations [39, 49]. Nevertheless, it is still possible that other mutations present in *CDK12*‐mutant tumours contribute to DNA methylation changes. To confirm that decrease in CDK12 activity can affect DNA methylation, we used both inhibitor‐ and knockdown‐based strategies. First, because transcription elongation can be supported by CDK12 and also CDK13, we identified an inhibitor that can target both, THZ531 [36]. Indeed, inhibition of CDK12 and CDK13 using THZ531 led to a significant decrease in DNA methylation (Figure 2C). Second, to validate the importance of decreased CDK12 activity in lowering DNA methylation, we performed MeDIP qPCR experiment after knockdown of CDK12 in two models of CRPC. This experiment revealed that knockdown of CDK12 significantly decreases DNA methylation; however, we note that dual inhibition of both CDK12 and CDK13 exerts stronger impact (Figures 2D and S2). Nevertheless, our results provide evidence linking *CDK12* mutations in patient samples to decrease in DNA methylation and show that simultaneous targeting of CDK12 and CDK13 in vitro decreases DNA methylation across the genome. To our knowledge, this is the first time CDK12 or CDK13 has been associated with altered DNA methylation.

An outstanding question emerging from our experiments is the mechanistic basis of the decreased DNA methylation when CDK12 and/or CDK13 activity decreases. In principle, the decrease in DNA methylation may either be passive resulting from decreased activity of DNA methyl transferases or active, through the action of the TET dioxygenases [50, 51, 52]. But it remains unclear how these factors are either prevented from associating with chromatin or their association is increased when CDK12/13 activity decreases. An obvious candidate in mediating the differential association with chromatin is the transcription machinery, in particular the transcriptionally active RNA Pol II itself, which should be particularly affected when CDK12/13 is inhibited [53, 54] (Figure 5B). Here, we also want to highlight an alternative mechanism affecting DNA methylation, as during DNA replication the appropriate methylation pattern is established on the newly replicated strand [55]. Decrease in CDK12 activity has been shown to lead to conflicts between transcription and replication machineries [12]. In the future, it is therefore imperative to map the composition of DNA replication forks using techniques such as ‘isolation of proteins on nascent DNA’ (iPOND) in cells with defective CDK12 to establish if effects on DNA methylation can be explained through recruitment of the DNA methylation machinery to replication forks.

We propose that decrease in DNA methylation is a stochastic process and if decrease in DNA methylation occurs in loci that confer proliferative advantage, these cells go on to establish the tumour. According to this proposal, we show that DNA methylation is significantly depleted from the MYC binding sites both in patient tumours with *CDK12* inactivation and in response to dual inhibition of CDK12/13 in vitro (Figure 3B,C). Based on the literature, decrease in CDK12 activity increases MYC expression also through direct effects on RNA Pol II activity: Decrease in CDK12 activity stimulates transcription of the short mRNAs, and MYC itself is a short mRNA stimulated in this situation [42, 56]. Mechanistically, decrease in CDK12 activity leads to decrease in the interaction between CDK9 and its endogenous inhibitor, which explains stimulation of transcription of the short genes [19]. Our findings and the existing literature propose that decrease in CDK12/13 activity decreases DNA methylation, which together with effects on transcriptionally active RNA Pol II, lead to upregulation of signalling through oncogenic transcription factors such as MYC. We propose that stochastic decrease in DNA methylation together with increased MYC expression leads to increased plasticity of cancer cells, which promotes their metastatic potential. In the future, it should be assessed if depletion of MYC prevents the pro‐tumorigenic phenotype brought about due to decrease in CDK12 activity.

Aberrant activation of MYC directly induces replication stress through multiple mechanisms, thereby compromising genomic stability and potentially contributing to *CDK12*‐inactivation driven phenotype. Previously, it has been established that MYC directly regulates the transcription of DNA replication initiation factors, leading to excessive activation of replication initiation and increasing the likelihood of transcription/replication conflicts [57]. The uncontrolled replication initiation can result in replication fork stalling and collapse, which subsequently trigger the DNA damage response [58]. In addition to controlling transcription of factors involved in DNA repair, MYC has also been shown to remodel the CTCF‐mediated chromatin architecture. Remodelling the three‐dimensional genome may further exacerbate conflicts between transcription and replication, thereby elevating levels of replication stress [59]. Accordingly, MYC‐driven proliferation forces cancer cells to become overly dependent on DNA damage repair pathways for survival [60]. In this situation, high activity of CDK12 should in principle be of high importance to support the robust expression of genes involved in DNA repair, and it is therefore unexpected that decrease in CDK12 activity augments MYC‐driven transcription and proliferation.

The already established role of CDK12 in maintaining the expression of genes required for homologous recombination, and MYC as a factor that induces replication stress prompted us to ask if prostate cancer cells become dependent on DNA‐PK when they acutely lose CDK12/13 activity. Indeed, depletion of CDK12 activity either using siRNAs or small molecule inhibitor‐based approaches targeting both CDK12/13 sensitize cancer cells to DNA‐PK inhibition (Figure 4B–D). Earlier, decrease in CDK12 activity has been shown to cause sensitivity against PARP inhibitors [8, 61]. CDK12 is important for the transcription of genes involved in DNA damage response [62], which can explain why cancer cells become more dependent on the kinase to tolerate genotoxic stress. Mechanistically, we show that DNA‐PK is required to promote MYC‐dependent transcription in cells that have decreased activity of CDK12/13 (Figure 5D). In the future, it is important to establish how DNA‐PK affects MYC‐driven transcription and other cellular processes.

In summary, here we show that the decreased activity of transcription elongation kinases CDK12 and CDK13 regulates the epigenetic state of cancer cells and leads to decrease in DNA methylation from MYC target genes. In this situation, cells become more dependent on DNA‐PK, and co‐targeting DNA‐PK and CDK12/13 leads to combinatorial lethality. We propose that this interaction opens an actionable therapeutic vulnerability.

## 5 Conclusion

In this study, we show that decrease in CDK12 activity lowers DNA methylation to support MYC‐dependent transcription. In support of the role of CDK12 in the expression of factors involved in homologous recombination, we show that decrease in CDK12 activity sensitizes cancer cells to DNA‐PK inhibition. Our study identifies an epigenetic adaptation to decrease in CDK12/13 activity and proposes *CDK12* inactivation as a biomarker of sensitivity to DNA‐PK inhibitors.

## Author Contributions

**Harri M. Itkonen:** conceptualization, methodology, investigation, formal analysis, supervision, funding acquisition, project administration, resources, writing – original draft, writing – review and editing, visualization. **Aishwarya Gondane:** formal analysis, investigation, funding acquisition, writing – original draft, writing – review and editing. **Jing Liang:** methodology, software, data curation, investigation, validation, formal analysis, funding acquisition, visualization, writing – original draft, writing – review and editing.

## Funding

J.L. was supported for a part of this project by funding from the Maud Kuistila Memorial Foundation, Biomedicum Helsinki Foundation and Doctoral Education Pilot in Precision Cancer Medicine in University of Helsinki. A.G. is supported in part by The Magnus Ehrnrooth foundation and the Young investigator grant from Biomedicum. H.M.I. is grateful for the funding from the Academy of Finland (Decision nrs. 331324, 358112 and 335902), the Jenny and Antti Wihuri Foundation, the Cancer Foundation Finland and the Sigrid Juselius Foundation. The funders had no role in the conceptualization, design, data collection, analysis, decision to publish, or preparation of the manuscript.

## Conflicts of Interest

The authors declare no conflicts of interest.

## Supporting information


**Figure S1:** Truncating mutations in the *CDK12*‐ gene and alterations in the *IDH1*‐gene are mutually exclusive in prostate cancer patient samples. The plot was generated using the tools available in cBioPortal and the data is from the TCGA dataset (Firehose Legacy). Each vertical bar along the x‐axis represents an individual patient sample. Coloured bars indicate the presence of specific genetic alterations, including truncating mutations (putative driver, black), deep deletions (blue), missense mutations (putative driver, dark green; unknown significance, light green) and elevated gene expression (mRNA High, pink). The solid grey bars represent patient samples with no detectable genetic alterations in these respective genes. Please, note that there are altogether 491 samples but as these all do not have alterations in the genes of interest, they are not shown in this figure.
**Figure S2:** Knockdown of CDK12 alone causes locus‐specific decrease in DNA methylation. (A) The selective enrichment of methylated DNA after MeDIP was validated using qPCR in 22RV1 and C4‐2 cells with CDK12 knockdown (siCDK12_1 and siCDK12_2). Cells were collected 3 days after CDK12 knockdown for MeDIP experiments. Promoter of *TDRD1* serves as hypermethylated positive control, while promoters of transcriptionally active *AR* and *MYC* act as unmethylated negative controls. (B) MeDIP‐qPCR evaluation of DNA methylation at the four selected candidate genes (*DPF1*, *NR125885.1*, *PLAC8* and *RTN4L1*) following CDK12 knockdown in both 22RV1 and C4‐2 cell lines. Data demonstrates a statistically significant decrease in methylation specifically on the *DPF1*‐gene, highlighting its distinct sensitivity to CDK12 depletion. Student's *t*‐test was used to assess the significance (*n* = 3 biological replicates; *p < 0.05, **p < 0.01, ***p < 0.001).
**Figure S3:** Validation of combinatorial lethality between inhibitors of CDK12/13 (THZ531) and DNA‐PK (AZD7648). Colony formation assay of 22RV1 cells in one week after the indicated treatments (AZD7648: 1 μM).
**Figure S4:** Knockdown of DNA‐PK sensitizes 22RV1 cells to CDK12/13 inhibitor THZ531. Left panel: Confirmation of DNA‐PK knockdown using western blotting. Densitometry analysis of DNA‐PK protein levels relative to Actin confirms its successful knockdown (cells treated for 3 days; *n* = 3 biological replicates). Statistical significance was determined using *t*‐test. Right panel: Cell viability of 22RV1 line after 4 days of DNA‐PK knockdown with CDK12/13 inhibition (THZ531) for the last three days. The data is from four biological replicates (each having three technical replicates) with SEM, and ANOVA was used to assess the significance. Faint vertical bars visually separate different treatment doses of CDK12/13 inhibitor.
**Figure S5:** Knockdown of CDK12 sensitizes 22RV1 cells to DNA‐PK inhibitor AZD7648. Left panel: Confirmation of CDK12 knockdown using western blotting. Densitometry analysis of CDK12 protein levels relative to Actin confirms its successful knockdown (cells treated for 3 days; *n* = 3 biological replicates). Statistical significance was determined using *t*‐test. Right panel: Cell viability of 22RV1 cell line after 4 days of CDK12 knockdown with DNA‐PK inhibition (AZD7648) for the last three days. The data is from four biological replicates (each having three technical replicates) with SEM and ANOVA was used to assess the significance. Faint vertical bars visually separate different treatment doses of DNA‐PK inhibitor.
**Figure S6:** Co‐inhibition of DNA‐PK (AZD7648) and CDK12/13 (THZ531) displays synergistic activity in prostate and breast cancer cell lines. Evaluation of synergistic interaction between inhibition of CDK12/13 (THZ531) and DNA‐PK (AZD7648) using SynergyFinder 2.0 [63]. Data is representative of two biological replicates.
**Figure S7:** DNA‐PK (AZD7648) inhibition augments CDK12/13 inhibitor‐induced effects on RNA Pol II phosphorylation before cell death in 22RV1 cell line. 22RV1 cells were treated with DNA‐PK inhibitor (20 μM AZD7648), CDK12/13 inhibitor (100 nM THZ531, referred to as CDK12i in the figure) or combination of the two for 4 h and 24 h, and samples were analysed using western blot. Densitometry was used to record signal intensity and the abundance of the RNA Pol II p‐Ser‐2 and PARP proteins are presented relative to control (control is set to 1).
**Figure S8:** MYC target V1 and V2 gene sets are the most significantly downregulated sets with combined inhibition of CDK12/13 (THZ531) and DNA‐PK (AZD7648). Differentially expressed genes (standard RNA‐seq analysis) between untreated and 20 μM AZD7648 treatment, untreated and 100 nM THZ531 treatment, untreated and combination of AZD7648 and THZ531 treatment were identified and subjected to GSEA using fgsea. MYC target V1 and V2 gene sets are highlighted in red.

## Data Availability

The datasets supporting the conclusions of this article are included within the article and its additional files. SLAM‐ and MeDIP‐seq data have been deposited to GEO‐database with accession numbers GSE287819 and GSE287617.
