# Supplementary material for: CDK12/13 Inhibition Induces DNA Hypomethylation and Sensitizes Cancer Cells to Compounds Targeting DNA‐PK
Source: J Cell Mol Med. 2026 Aug 20;30(16):e71323. doi: 10.1111/jcmm.71323 (PMC13494145; doi:10.1111/jcmm.71323)
Supplement: Supplementary file 1 [file JCMM-30-e71323-s001.docx]

**Supplementary figures and figure legends**

**Supplementary figure 1. Truncating mutations in the *CDK12*- gene and alterations in the *IDH1*-gene are mutually exclusive in prostate cancer patient samples.** The plot was generated using the tools available in cBioPortal and the data is from the TCGA dataset (Firehose Legacy). Each vertical bar along the x-axis represents an individual patient sample. Colored bars indicate the presence of specific genetic alterations, including truncating mutations (putative driver, black), deep deletions (blue), missense mutations (putative driver, dark green; unknown significance, light green), and elevated gene expression (mRNA High, pink). The solid grey bars represent patient samples with no detectable genetic alterations in these respective genes. Please, note that there are altogether 491 samples but as these all do not have alterations in the genes of interest, they are not shown in this figure.

**Supplementary figure 2**. **Knockdown of CDK12 alone causes locus-specific decrease in DNA methylation.** **A)** The selective enrichment of methylated DNA after MeDIP was validated using qPCR in 22RV1 and C4-2 cells with CDK12 knockdown (siCDK12_1 and siCDK12_2). Cells were collected 3 days after CDK12 knockdown for MeDIP experiments. Promoter of *TDRD1* serves as hypermethylated positive control, while promoters of transcriptionally active *AR* and *MYC* act as unmethylated negative controls. **B)** MeDIP-qPCR evaluation of DNA methylation at the four selected candidate genes (*DPF1*, *NR125885.1*, *PLAC8*, and *RTN4L1*) following CDK12 knockdown in both 22RV1 and C4-2 cell lines. Data demonstrates a statistically significant decrease in methylation specifically on the *DPF1*-gene, highlighting its distinct sensitivity to CDK12 depletion. Student’s t-test was used to assess the significance (n=3 biological replicates; *p < 0.05, **p < 0.01, ***p < 0.001).

**Supplementary figure 3. Validation of combinatorial lethality between inhibitors of CDK12/13 (THZ531) and DNA-PK (AZD7648).** Colony formation assay of 22RV1 cells in one week after the indicated treatments (AZD7648: 1 μM).

**Supplementary figure 4. Knockdown of DNA-PK sensitizes 22RV1 cells to CDK12/13 inhibitor THZ531.** Left panel**:** Confirmation of DNA-PK knockdown using western blotting. Densitometry analysis of DNA-PK protein levels relative to Actin confirms its successful knockdown (cells treated for 3 days; n = 3 biological replicates). Statistical significance was determined using t-test. Right panel: Cell viability of 22RV1 line after 4 days of DNA-PK knockdown with CDK12/13 inhibition (THZ531) for the last three days. The data is from four biological replicates (each having three technical replicates) with SEM, and ANOVA was used to assess the significance. Faint vertical bars visually separate different treatment doses of CDK12/13 inhibitor.

**Supplementary figure 5. Knockdown of CDK12 sensitizes 22RV1 cells to DNA-PK inhibitor AZD7648.** Left panel**:** Confirmation of CDK12 knockdown using western blotting. Densitometry analysis of CDK12 protein levels relative to Actin confirms its successful knockdown (cells treated for 3 days; n = 3 biological replicates). Statistical significance was determined using t-test. Right panel: Cell viability of 22RV1 cell line after 4 days of CDK12 knockdown with DNA-PK inhibition (AZD7648) for the last three days. The data is from four biological replicates (each having three technical replicates) with SEM, and ANOVA was used to assess the significance. Faint vertical bars visually separate different treatment doses of DNA-PK inhibitor.

**Supplementary figure 6. Co-inhibition of DNA-PK (AZD7648) and CDK12/13 (THZ531) displays synergistic activity in prostate and breast cancer cell lines.** Evaluation of synergistic interaction between inhibition of CDK12/13 (THZ531) and DNA-PK (AZD7648) using SynergyFinder 2.0 [64]. Data is representative of two biological replicates.

**Supplementary figure 7. DNA-PK (AZD7648) inhibition augments CDK12/13 inhibitor-induced effects on RNA Pol II phosphorylation before cell death in 22RV1 cell line.** 22RV1 cells were treated with DNA-PK inhibitor (20 μM AZD7648), CDK12/13 inhibitor (100 nM THZ531, referred to as CDK12i in the figure) or combination of the two for 4 hours and 24 hours, and samples were analyzed using western blot. Densitometry was used to record signal intensity and the abundance of the RNA Pol II p-Ser-2 and PARP proteins are presented relative to control (control is set to 1).

**Supplementary figure 8. MYC target V1 and V2 gene sets are the most significantly downregulated sets with combined inhibition of CDK12/13 (THZ531) and DNA-PK (AZD7648).** Differentially expressed genes (standard RNA-seq analysis) between untreated and 20 μM AZD7648 treatment, untreated and 100 nM THZ531 treatment, untreated and combination of AZD7648 and THZ531 treatment were identified and subjected to GSEA using fgsea. MYC target V1 and V2 gene sets are highlighted in red.
